# Natural Killer Cells in Antifungal Immunity

**DOI:** 10.3389/fimmu.2017.01623

**Published:** 2017-11-22

**Authors:** Stanislaw Schmidt, Lars Tramsen, Thomas Lehrnbecher

**Affiliations:** ^1^Division for Pediatric Hematology and Oncology, Hospital for Children and Adolescents, Johann Wolfgang Goethe-University, Frankfurt, Germany

**Keywords:** natural killer cell, invasive fungal infection, *Aspergillus*, *Candida*, mucormycete, *Cryptococcus*, antifungal host response

## Abstract

Invasive fungal infections are still an important cause of morbidity and mortality in immunocompromised patients such as patients suffering from hematological malignancies or patients undergoing hematopoietic stem cell transplantion. In addition, other populations such as human immunodeficiency virus-patients are at higher risk for invasive fungal infection. Despite the availability of new antifungal compounds and better supportive care measures, the fatality rate of invasive fungal infection remained unacceptably high. It is therefore of major interest to improve our understanding of the host–pathogen interaction to develop new therapeutic approaches such as adoptive immunotherapy. As experimental methodologies have improved and we now better understand the complex network of the immune system, the insight in the interaction of the host with the fungus has significantly increased. It has become clear that host resistance to fungal infections is not only associated with strong innate immunity but that adaptive immunity (e.g., T cells) also plays an important role. The antifungal activity of natural killer (NK) cells has been underestimated for a long time. *In vitro* studies demonstrated that NK cells from murine and human origin are able to attack fungi of different genera and species. NK cells exhibit not only a direct antifungal activity *via* cytotoxic molecules but also an indirect antifungal activity *via* cytokines. However, it has been show that fungi exert immunosuppressive effects on NK cells. Whereas clinical data are scarce, animal models have clearly demonstrated that NK cells play an important role in the host response against invasive fungal infections. In this review, we summarize clinical data as well as results from *in vitro* and animal studies on the impact of NK cells on fungal pathogens.

## Introduction

Invasive fungal infections are still associated with significant morbidity and mortality. For example, a retrospective cohort study in the US demonstrated that as compared to patients without invasive aspergillosis, patients suffering from the infection had a significant longer hospital stay, caused significantly higher costs, and, most importantly, had a significant higher mortality (1). A population-based analysis of invasive fungal infections in France revealed that between 2001 and 2010, the incidence of invasive fungal disease due to *Candida* spp., *Aspergillus* spp., and mucormycetes increased by 7.8, 4.4, and 7.3% per year, respectively, which was highly significant for each pathogen (2). In contrast to cryptococcosis, which often occurs in human immunodeficiency virus (HIV)-patients, the population at high risk for candidemia, invasive aspergillosis, and mucormycosis includes in particular patients with hematological malignancies, patients undergoing hematopoietic stem cell transplantation (HSCT) and solid organ recipients (2–6). These patient populations are characterized by the impairment of multiple arms of the immune system (7, 8), such as of natural barriers, the phagocyte system, innate immunity, and lymphocytes, all of which may increase the risk for an invasive fungal infection. Therefore, it is not surprising that the mortality rate of invasive fungal disease is extremely high in these patient populations, exceeding 70% in HSCT recipients suffering from invasive aspergillosis or mucormycosis (4).

It is well known that the recovery of the immune system has a major impact on the outcome of invasive fungal infection in an immunocompromised patient (9, 10). Unfortunately, to date, immunomodulation using cytokine and growth factor therapies, as well as adoptive immunotherapeutic strategies such as granulocyte transfusions or the administration of *Aspergillus*-specific T-cells did not significantly improve the prognosis of immunocompromised patients with invasive fungal disease (11). It is therefore of major interest to improve our understanding of the host–pathogen interaction to develop new therapeutic strategies for immunocompromised individuals suffering from fungal infection. This review will summarize available clinical data as well as results from *in vitro* and animal studies on the impact of natural killer (NK) cells on fungal pathogens.

## The Host Response to Fungal Infection

Over the last decades, we could witness major advances not only in the understanding of the complexity of the immune system but also in our knowledge on the immunopathogenesis of invasive fungal infections. The host response to a fungal pathogen includes, but is not restricted to various cells of the innate and adaptive immunity such as monocytes, neutrophils, dendritic cells (DCs), T and B lymphocytes, as well as multiple soluble molecules such as collectins, defensins, cytokines including interferons (IFNs) (12, 13). Although it is known for a long time that severe and prolonged neutropenia (e.g., absolute neutrophil count ≤500/μl and duration of neutropenia ≥10 days) is the single most important risk factor for invasive aspergillosis, invasive *Candida* infection, and mucormycosis in patients receiving cytotoxic chemotherapy or undergoing allogeneic HSCT (9, 14), recent studies refined our understanding how neutrophils are controlling in particular the early stages of invasive fungal infection. Neutrophils are attracted by cytokines released by endothelial cells and macrophages and are able to quickly migrate to a focus of infection. In addition to recruiting and activating other immune cells by the production of pro-inflammatory cytokines, neutrophils may attack as front-line defense invading pathogens by phagocytosis, the production of reactive oxygen intermediates, and the release antimicrobial enzymes to the formation of complex extracellular traps (NETs) that help in the elimination of the fungus (15). DCs transport fungal antigens to the draining lymph nodes, where they orchestrate T cell activation and differentiation (16). A number of lymphocyte subsets have an important impact in the antifungal immunity, such as Th1 cells (important for inflammation and fungal clearance), Th17 cells (neutrophil recruitment, defensins), Th22 cells (defensins, tissue homeostasis), and Treg cells (immunosuppression). In addition, a number of cytokines play important roles in the complex crosstalk between different cells of the immune system, which modify and regulate innate and adaptive immune responses, such as the induction of proliferation and differentiation, as well as the activation or suppression of different target cells (11–13). Still, many open questions have to be resolved, including the influence of the genetic background in the delicate interplay of immune cells, the interaction of the innate and adaptive immune system in balancing protection and immunopathology in fungal infections (12), and the influence of fungal microbiota or “mycobiota” on health and disease (17). More importantly, we have to learn how to modify the immune system in the combat against invasive fungal infections, in particular in the immunocompromised host, which includes not only the activation of the immune response to eliminate the pathogen but also its suppression to avoid collateral tissue damage.

## NK Cell Biology

Human NK cells, which originate from the bone marrow, represent up to 15% of peripheral blood mononuclear cells. They are characterized by the expression of CD56 and by the absence of the T cell marker CD3. According to the surface expression density of CD56 and CD16, NK cells can be subdivided in two main subpopulations, namely the cytotoxic CD56^dim^CD16^bright^ and the immune regulatory CD56^bright^CD16^dim^ subsets (18). Although NK cells were originally considered as cells of innate immunity, they demonstrate qualities of the adaptive immunity such as immunological memory (19–22). In this regard, animal models and human studies indicate that NK cells are able to develop long-lasting antigen-specific memory (23–26). It has been demonstrated that memory-like NK cells display a less differentiated phenotype in CD56^dim^ NK cells, which were CD94^+^NKG2A^+^ but CD57^−^KIR^−^ (27). The name “natural killer cell” originally came from their ability to kill tumor cells *in vitro* and *in vivo* without previous stimulation (22, 28–31). Their antitumor activity includes activity against acute lymphoblastic leukemia (32), acute myeloid leukemia (33), and neuroblastoma (34, 35). In addition to their antitumor activity, NK cells play an important role in the host response against various pathogens which includes viruses such as cytomegalovirus (CMV), Epstein–Barr virus (23, 36–38), or hepatitis B and C virus (37, 39, 40), and Gram-positive, Gram-negative, and intracellular bacteria, such as *Salmonella typhi, Escherichia coli* (41), or *Listeria monocytogenes* (42).

Natural killer cells eliminate their potential targets either by directly using cytotoxic molecules such as perforin or granzyme B, which are stored in granules, or by death receptor-mediated apoptosis (36). In addition, CD16 (FcγRIII) triggers antibody-dependent cell-mediated cytotoxicity on opsonized target cells (36). Education and differentiation are considered to be important mechanisms for both direct and antibody-dependent functionality of NK cells (43–45). Several models have been developed to explain the process of “education” (43, 45–49). In general, the expression of self-recognizing inhibitory receptors (SRIR) results in the development of NK cells toward fully functional mature form, which has been termed as “licensing” process (50). In contrast, the “disarming” model describes NK cells lacking SRIR that become anergic due to chronic activation (51). The more dynamic “rheostat model” has been used to describe that stronger inhibitory signaling through more SRIR interactions results in a greater functional responsiveness of NK cells (52, 53). Importantly, cytokine stimulation can prime SRIR-deficient NK cells to a functional state (50), and uneducated cells are also able to combat viral infections, as SRIR-deficient NK cells strongly respond toward murine CMV (54).

In addition to the direct cytotoxic abilities, NK cells have recently been classified closely to group 1 innate lymphoid cells, which are characterized by the production of IFN-γ, whereas type 2 cytokines are not produced (55). *Via* the release of chemokines and cytokines such as IFN-γ, tumor necrosis factor alpha (TNF-α), granulocyte-macrophage colony-stimulating factor (GM-CSF), or chemokine ligand 5 (CCL5) [regulated upon activation, normal T-cell expressed, and secreted (RANTES)], NK cells modulate the activity of various immune cells including neutrophils, DCs, and T cells (46, 56), which complements their direct anti-pathogen and antibody-mediated activities.

## NK Cells and Invasive Fungal Infection: Clinical Observations

Although clinical data suggest the importance of NK cells in the risk and outcome of invasive fungal infection, the exact role of NK cells is difficult to determine as multiple cells are involved in the antifungal host response, and as these cells interact in a complex network with both positive and negative feedback mechanisms (7, 8). A recent study analyzed 51 patients undergoing allogeneic HSCT, among them 9 patients in whom proven or probable invasive aspergillosis occurred (10). The study evaluated both the quantitative and qualitative reconstitution of immune cells including polymorphonuclear cells, CD4^+^ T cells, CD8^+^ T cells, and NK cells, and the authors reported two important observations: first, transplant recipients suffering from invasive aspergillosis displayed insufficient NK cell recovery with cell counts remaining less than 200/μl as well as lower reactive oxygen species (ROS) production. Second, HSCT transplant recipients who were cured from invasive aspergillosis had significantly higher ROS production and higher NK cell counts as compared to those patients who had a poor outcome of the invasive fungal infection. As both cell count and ROS production were altered in each of the analyses, the importance of the NK cell count as single risk and single prognostic factor for invasive aspergillosis remains unresolved. Another study evaluated 396 patients undergoing solid organ transplantation (57). A total of 304 patients were kidney and 92 patients were liver transplant recipients, and median followed-up time was 504.5 days after transplantation. The analysis demonstrated that 1 month after transplantation, patients who did not develop invasive fungal disease at a later time point had significantly higher mean NK cell count as compared with those patients who developed fungal disease. In the non-transplant setting, larger clinical studies on the impact of NK cell-mediated immunity on fungal infections are lacking. Although it was observed that patients suffering from chronic mucocutaneous candidiasis (CMCC) have a decrease of both NK cell number and cytotoxic activity (58–60), the exact impact of NK cells in the pathogenesis of the infection or in the progression of the disease is hard to define, in particular as cell-mediated immunity is also impaired in patients with CMCC. Although it may very well be that the pathologic NK cell findings are a risk factor for CMCC, other possible explanations for the decreased NK cell count and NK cell activity include that the fungus had a negative impact on originally normal NK cells, or that the observation is an epiphenomenon only. Similarly, it was reported that a patient developed a *Trichophyton rubrum* infection during corticosteroid treatment for systemic lupus erythematosus (61). Although immunosuppressive therapy was stopped, the infection remained. Further evaluation demonstrated that both numbers and activity of NK cells were reduced. The authors speculated that the impairment of the NK cells was causing the infection, but again, one could also argue that the infection resulted in a decreased NK cell number and NK cell activity in this individual patient.

## NK Cells Damage Various Fungi *In Vitro*

**Figure 1:**
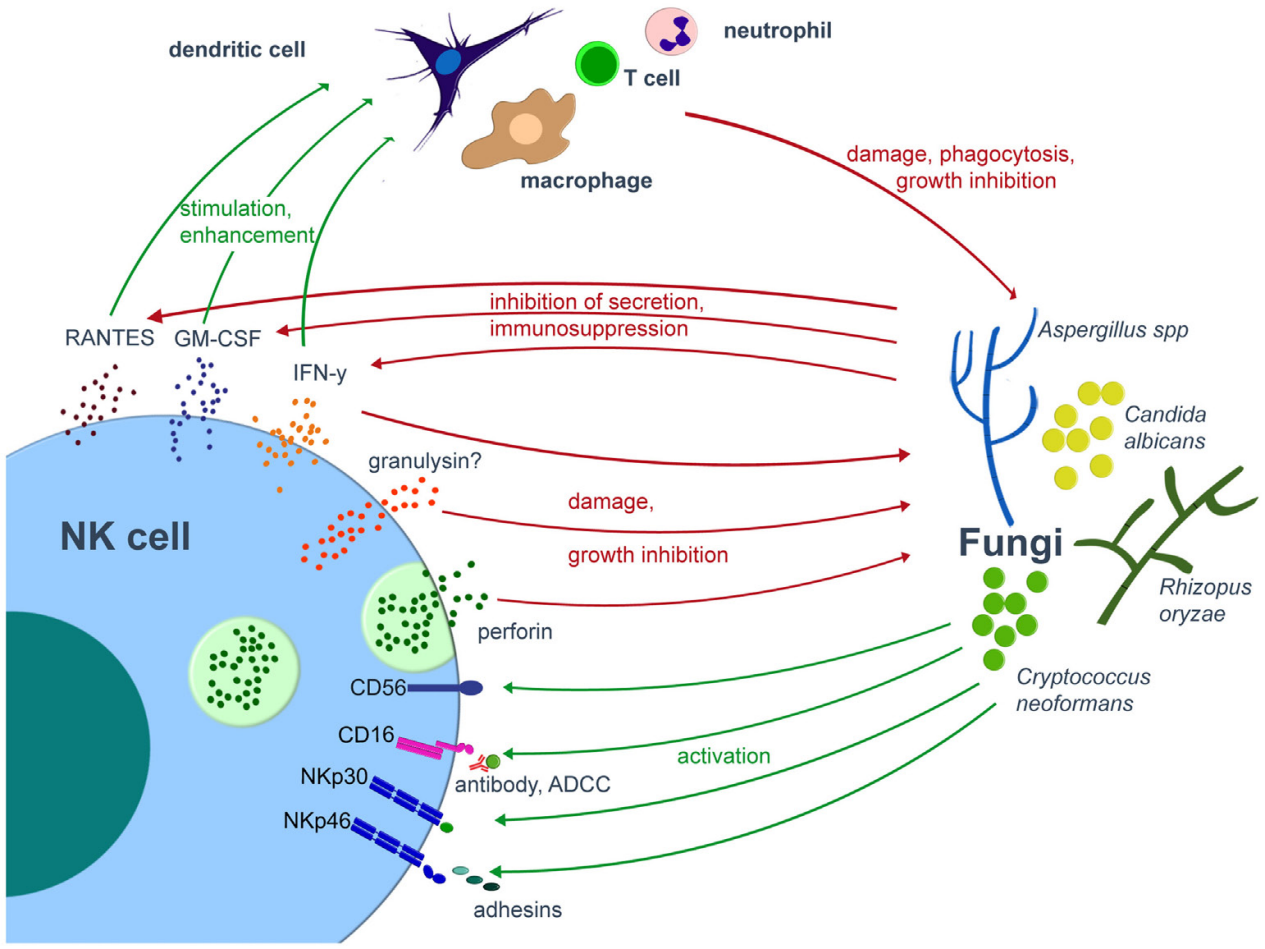
Interplay of NK cells and fungal pathogens. Various fungal pathogens are able to activate NK cells. Once activated, NK cells directly damage fungi by cytotoxic molecules such as perforin or release cytokines, by which they modulate the antifungal host response *via* various immune cells. On the other hand, the fungus may compromise the host immune system. Green arrows indicate activation/stimulation, red arrows inhibition/damage. ADCC, antibody-dependent cell mediated cytotoxicity; IFN, interferon; GM-CSF, granulocyte-macrophage colony-stimulating factor; NK, natural killer.

Multiple studies published over the last three decades demonstrate that both murine and human NK cells exhibit antifungal activity *in vitro* against various fungal pathogens, such as *Aspergillus fumigatus, Aspergillus niger, Candida albicans, Cryptococcus neoformans, Paracoccidioides brasiliensis, Rhizopus oryzae*, and other mucormycetes including *Lichthemia ramosa* or *Absidia corymbifera* (62–70) (Figure 1). NK cells damage the hyphal form of *A. fumigatus* and *R. oryzae*, but are not able to exhibit fungicidal activity toward conidia (62, 63, 65). In *C. albicans*, human NK cell are cytotoxic against germ tubes and additionally are able to phagocyte *C. albicans* yeasts (8 ± 0.5% of *C. albicans* yeasts were phagocytosed by NK cells within the first 2 h of interaction) (70). The lack of antifungal activity against conidia may be explained by the fact that conidia are often protected by capsules, by pigments such as melanin, or by hydrophobic layers, all of which may prevent recognition by various immune cells (71–74). For example, the rodlet/hydrophobin layer on dormant *A. fumigatus* conidia masks the recognition by the immune system and thus prevents an host immune response, whereas the genetical removal the rodlet/hydrophobin layer in dormant conidia of the *ΔrodA* mutant resulted in the induction of maturation and activation of human DCs (71). Similarly, the lack of a capsule in the strain CAP67 of the yeast-like fungus *C. neoformans* leads to a higher expression of the cytotoxic molecule perforin by NK cells as compared to the encapsulated strain B3501 (75).

## Recognition of Fungi by NK Cells

Over the last years, there were major advances in the identification and characterization of receptors by which NK cells recognize fungal pathogens (Figure 1, Table 1). NK cells express unique activating receptors on their surface, which are called natural cytotoxicity receptors (NCR) 1–3 (NKp46, NKp44, and NKp30; CD335–CD337). Studies using blocking antibodies and siRNA to knockdown the NKp30 expression on the surface of the cell line YT demonstrated that NK cells are able to directly recognize *C. albicans* and *C. neoformans* by the NKp30 receptor, which further mediates killing of these fungi (68). However, polymorphonuclear neutrophils and granulocyte myeloid-derived suppressor cells may decrease the NKp30 expression on NK cells, which results in reduced cytotoxicity toward *A. fumigatus* and a decrease in IFN-γ secretion (76). Recently, the NKp46 receptor and its mouse ortolog NCR1 were identified to play an important role in the NK cell-mediated killing of *C. glabrata* (77). It was speculated whether NKp46/NCR1 may be a novel type of pattern recognition receptor, as these receptors not only recognize the *C. glabrata* adhesins Epa1, Epa6, and Epa7 but also bind viral adhesion receptors (77). The importance of the receptor is underlined by the observation that NCR1-deficient mice were unable to clear *C. glabrata* systemic infection (77). Because several fungi including *Aspergillus, Cryptococcus*, and *Coccidioides* express adhesins (78), further studies have to evaluate whether and to what extent these fungal adhesins are recognized by which of the NK cell receptors (79).

**Table 1:** Natural killer (NK) cell receptors in antifungal response.

Receptor	Origin	Ligand	Fungus	Remarks	Reference
NKp30	Human, YT cell line	Undefined pathogen-associated molecular pattern	*Candida albicans, Cryprococcus neoformans*	NKp30 required for YT cell cytotoxicity toward fungal pathogens *in vitro* siRNA knockdown of NKp30 results in decreased release of perforin Addition of anti-NKp30 antibody or inhibition of NKp30 via siRNA results in decreased antifungal activity	Li et al. (68)

NKp46	Human	Fungal adhesins Epa1, Epa6, Epa7	*Candida glabrata*	Human NKp46 and mouse ortholog NCR1 bind *C. glabrata in vitro*	Vitenshtein et al. (77)
NCR1	Mouse			Clearing of systemic *C. glabrata* infection *in vivo* depends on recognition of fungal adhesins by NCR1	

CD56	Human	Unknown	*Aspergillus fumigatus*	Blocking of CD56 by inhibitory antibodies reduces fungal-mediated NK cell activation and inhibits amount of secreted cytokines *in vitro*	Ziegler et al. (80)

CD16	Mouse	Cryptococcal polysaccharide	*Cryptococcus neoformans*	Purified IgG fraction of rabbit anticryptococcal antibody augments growth inhibitory activity of murine splenic NK cells *in vitro*	Nabavi and Murphy (81)

Recent studies suggested CD56 as pathogen recognition receptor, as it was demonstrated by flow cytometry that the fluorescence positivity of the surface receptor significantly decreased upon fungal contact (80). The authors could visualize the direct interaction of NK cells and *A. fumigatus via* CD56, which was re-organized and accumulated at this interaction site time dependently. Importantly, blocking of CD56 surface receptor reduced fungal-mediated NK cell activation and reduced cytokine secretion. Earlier studies have demonstrated that the low-affinity Fc-receptor CD16 [FcγRIIIa (CD16a) and FcγRIIIb (CD16b)] is also involved in the antifungal activity of NK cells, as NK cells inhibited the growth of *Cryptococci* more effectively in the presence of anti-cryptococcal IgG antibodies than in the presence of normal rabbit serum or medium (81). However, it is important to note that primary and pre-activated NK cells downregulate CD16 after contact with *C. albicans*, which has also been described for the cellular adhesin CD56 and immunoreceptor tyrosine-based activation motif-bearing receptors NKG2D (CD314) and NKp46 (CD335) (70). Taken together, we just begin to understand the complexity how NK cells are being activated by fungal pathogens.

## Direct Damage of Fungal Pathogens by NK Cells

Various mechanisms are described by which NK cells directly kill tumor cells, which include the release of soluble cytotoxic molecules such as perforin or granzyme, or the induction of apoptosis by the Fas–FasL or the TNF pathway. Regarding fungal pathogens, several studies reported on the importance of lytic granules released by NK cells. For example, the use of monensin, which inhibits granule secretion, partially abrogated the growth inhibition of *C. neoformans* by human NK cells [reviewed in Ref. (79)]. It further became clear that mainly perforin and granulysin mediate the direct NK cell cytotoxicity toward fungal pathogens (67, 82). When pretreating human NK cells with concanamycin A (ConA), which induces accelerated perforin degradation *via* an increase of pH in the lytic granules, significantly less damage of *A. fumigatus* and *R. oryzae* hyphae can be observed as compared to the addition of untreated NK cells to the fungus (62, 63, 83). Other studies used purified perforin and reported on fungal damage of *A. fumigatus* hyphae (62), the inhibition of filamentation of *C. albicans* (84), and the inhibition of the metabolic activity of *C. albicans* and *R. oryzae* in a dose-dependent manner (63, 70). The fact that ConA did not totally abrogate NK cell-mediated fungal damage suggests that other molecules than perforin also participate in the antifungal activity of human NK cells, as reported for *A. fumigatus, C. albicans*, and *R. oryzae*. Interestingly, inhibition of perforin by ConA or by small interfering RNA decreased NK cell anti-cryptococcal activity, whereas inhibition of granulysin did not alter the antifungal effect (67). However, it has been shown that the defective anti-cryptococcal activity of NK cells from HIV-patients can be corrected by *ex vivo* treatment with interleukin (IL)-12 (68), as IL-12 restores the lower perforin expression in NK cells from HIV-infected patients as well as the defective granule polarization in response to *C. neoformans* (85). In tumor cells, perforin perforates the membrane of the target, which leads to an influx of water and a loss of intracellular molecules, resulting in cell lysis (86, 87). Similarly, granulysin disrupts the target cell membrane, which results in higher intracellular calcium and lower intracellular potassium concentrations, both of which ultimately activate caspases and programmed cell death (apoptosis) (88–92). However, it is important to note that the mechanisms of the antifungal effect of perforin and granulysin have not fully been elucidated to date.

There is an ongoing controversy on the direct antifungal effect of IFN-γ. One study reported on a direct IFN-γ-mediated antifungal activity of NK cells against *Aspergillus*, which was independent of degranulation of NK cells and their cytotoxic molecules (65). The authors suggested as explanation that “IFN-γ might cooperate with fungal ribotoxins, (…), transforming them into suicide molecules for fungus” (65). Similarly, it was demonstrated that IFN-γ at a concentration of 32 pg/ml exhibited a small but significant antifungal effect on *A. fumigatus, A. flavus*, and *Saccharomyces cerevisiae*, and inhibited the growth by 6, 11, and 17%, respectively (93). As higher concentrations of IFN-γ, e.g., 50 or 100 pg/ml, did not increase antifungal activity, and IFN-γ serum levels of 18 ± 30 pg/ml can be detected in healthy individuals (94), the importance of the direct antifungal effect *in vivo* is questionable. Corroborating the data of another report (70), no significant antifungal effects of IFN-γ were detected in *Candida* and *C. neoformans* (93). However, it is important to note that a combination of amphotericin B at a concentration of 1 µg/ml and IFN-γ at 32 pg/ml increased the efficacy of amphotericin B against *A. fumigatus*, which might be important for immunotherapeutic strategies.

Inducing apoptosis *via* the Fas–FasL or the TNF pathway is another mechanism by which NK cells are able to kill a target and has been described for various tumor cells as well as for pathogen infected cells (95, 96). Whereas data on apoptosis are missing for molds, apoptosis in yeast cells has been reported, but molecular mechanisms at the core of apoptotic execution is still unknown (97, 98). One recent study reported that blocking the death receptor ligands FasL and tumor necrosis factor-related apoptosis inducing ligand on the surface of human NK cells by antibodies did not have any impact on the antifungal activity (70). In addition, phagocytosis may be another mechanism of direct fungal damage by NK cells, which has been reported for *C. albicans* yeast (70). Notably, the IgG fraction of rabbit anti-cryptococcal serum enhanced the anti-cryptococcal activity of NK cells *via* their CD16 receptor (81).

## Modulation of the Antifungal Host Response by NK Cells

Upon stimulation, NK cells produce various cytokines, all of which modulate the host immunity against fungi. IFN-γ is one of the key molecules in the antifungal host response and is constitutively produced by NK cells (99). IFN-γ exhibits multiple effects on various immune cells. For example, IFN-γ is able to stimulate migration, adherence, phagocytosis, as well as oxidative killing by neutrophils and macrophages. Conditioned medium from co-incubation of NK cells and *C. albicans* enhanced polymorphonuclear neutrophil activation (70). In addition, data of a murine model demonstrated the pivotal role of IFN-γ-producing NK cells in inducing the phagocytic activity of splenic macrophages, thus mediating protection against systemic infection with *C. albicans* (100). As NK cells are the main source of IFN-γ in neutropenic mice suffering from aspergillosis, depletion of NK cells resulted in diminished IFN-γ levels in the lungs followed by an increased fungal load (101). Interestingly, the fungal load could be reduced by the transfer of wild-type IFN-γ producing NK cells, whereas this was not seen when transferring NK cells from IFN-γ-deficient mice. Because IFN-γ also enhances maturation of DCs and plays a pivotal role in the protective T_H_1 cell response (11, 12, 102), the molecule was used as immunotherapy in invasive fungal disease. Whereas the administration of IFN-γ to mice with invasive aspergillosis was leading to reduced fungal burden and increased survival (103), available clinical data are inconclusive and do not allow a final conclusion on the usefulness of this strategy (11).

In addition to IFN-γ, NK cells produce soluble molecules such as GM-CSF and RANTES, both of which augment the host immune response *via* the stimulation of phagocytes and T cells, respectively (104–106).

## Interplay of NK Cells and Fungi

Fungi have developed strategies to counteract the complex and sophisticated antifungal immune response of the host. For example, *A. fumigatus* galactosaminogalactan induces apoptosis of polymorphonuclear neutrophils (107). Shedding of this molecule results in NK cell activation, which, in turn, leads to a Fas-dependent apoptosis-promoting signal in polymorphonuclear neutrophils (108). Galactosaminogalactan also induces IL-1 receptor antagonist, which leads to the suppression of IL-17 and IL-22 in peripheral blood mononuclear cells (109), and similar effects were observed with *Aspergillus* chitin (110). In addition, mycotoxins such as gliotoxin or aflatoxin (111) inhibit the phagocytic activity of macrophages, induce the apoptosis of monocytes, decrease the activation of nicotinamide adenine dinucleotide phosphate oxidase in neutrophils, and impair functional T cell responses (112–116), all of which hampers the host immune response toward the pathogen.

When NK cells are co-incubated with *A. fumigatus* or *R. oryzae*, lower levels of IFN-γ, GM-CSF, and RANTES are detected in the supernatant as compared to NK cells incubated alone (62, 63). Surprisingly, *A. fumigatus* increases the gene expression of IFN-γ in NK cells, but inhibits its release, thus leading to intracellular accumulation and decreased extracellular availability (117). Similarly, various mucormycetes affect the IFN-γ release by human NK cells (64). In contrast, earlier studies report that *C. neoformans* downregulates of the production of GM-CSF and TNF-α in unstimulated human NK cells, as assessed by gene expression and supernatant protein levels (118).

When looking at the fungal pathogen, it has been demonstrated that co-incubation of NK cells with *A. fumigatus* upregulated the expression of several stress-related fungal genes (117). This has been demonstrated for the heat shock protein *hsp90* or the ferric reductase *freB* (117). In *A. fumigatus*, Hsp90 plays an important role in the compensatory repair mechanisms of the cell wall in response to stress induced by antifungals, and Hsp90 has been described as a trigger for resistance to high concentrations of caspofungin, known as the paradoxical effect (119). FreB has recently been identified as an important enzyme in filamentous fungi which helps the fungus to adapt to iron starvation (120). Similarly, perforin-induced reduction of iron availability leads to the upregulation of the gene expression of *CSA2* in *C. albicans*, which is involved in the uptake of iron of human hemoglobin (84). Further characterization of specific interactions of the host immune system and fungal pathogens might identify novel targets for the antifungal armamentarium, e.g., the disruption of Hsp90 circuitry by Hsp90 inhibitors or anti-calcineurin drugs.

## NK Cells and Invasive Fungal Infection: Animal Studies

The few data of animal models clearly support the *in vitro* findings that NK cells play an important role in the antifungal host immune response. An early study in mice infected with *A. niger* demonstrated the association of the proliferation of NK cells and the inhibition of fungal growth (69). In addition, depletion of NK cells in mice inoculated with *C. neoformans* resulted in a considerably higher fungal load in the lungs as compared to untreated animals (121). In addition, antibody-mediated depletion of NK cells also decreased the phagocytosis of *C. albicans* by splenic macrophages as compared to controls (5.2 versus 21.5%) (100), and depletion of NK cells in mice *via* anti-asialo GM1 antibody resulted in enhanced susceptibility to *Histoplasma capsulatum* (122). Interestingly, in neutropenic mice, antibody-mediated depletion of NK cells also resulted in impaired clearance of the pathogen from the lungs and in a greater than twofold increase in mortality as compared to neutropenic mice with NK cells (123).

Importantly, the adoptive transfer of NK cells in mice lacking these cells can restore antifungal resistance. For example, in cyclophosphamide-pretreated mice suffering from cryptococcosis, the adoptive transfer of NK cell-enriched cell populations resulted in an enhanced clearance of the fungus as compared to controls receiving NK cell-depleted grafts (124, 125). As noted above, NK cell-derived IFN-γ plays an important role in the antifungal host response. NK cell depletion in neutropenic mice with invasive aspergillosis was leading to reduced lung IFN-γ levels and increased pulmonary fungal load, which was independent of T and B cell lymphocytes (101). However, the transfer of activated NK cells from wild-type, but not from IFN-γ-deficient mice resulted in better clearance of *A. fumigatus* from the lungs of both IFN-γ-deficient and wild-type recipients. Based on these findings, future studies have to assess in which clinical circumstances the adoptive transfer of NK cells to an immunocomoromised host suffering from an invasive fungal infection will be of benefit.

## Conclusion and Perspectives

There is increasing evidence that NK cells play an important role in the antifungal host response. *In vitro* data show that multiple fungal pathogens are able to activate NK cells, and further research will hopefully shed more light in the characterization of the complex interplay of NK cell receptors and fungal ligands. Once activated, NK cells directly damage the fungus by soluble cytotoxic molecules such as perforin, whereas the role of other mechanisms such as the induction of apoptosis *via* different pathways is still relatively unclear. In addition to the direct fungal damage, NK cells release multiple cytokines and IFNs by which they modulate the immune system, e.g., *via* neutrophils and T cells. As cure from an infectious complication not only depends on the successful activation of the immune system but also from a timely downregulation and resolution of the inflammatory process, further research needs to characterize the release of pro- and anti-inflammatory molecules. How and to what extent the fungus itself alters its gene expression profile in the presence of NK cells remains another research gap. In this regard, we have to learn how the fungus compromises the host immune system, which might offer new targets in our combat against the pathogen. In addition, animal studies will help to clarify the benefit and potential risks of using NK cells as adoptive preventive or therapeutic strategy, which may be a significant step toward decreasing morbidity and mortality of invasive fungal infection in the clinical setting.

## Author Contributions

All authors were involved in the concept of the manuscript, search and analysis of the references, and writing the manuscript; read and approved the final version of the manuscript.

## Conflict of Interest Statement

The authors declare that the research was conducted in the absence of any commercial or financial relationships that could be construed as a potential conflict of interest.

## Abbreviations

ADCC, antibody-dependent cell-mediated cytotoxicity; CD, cluster of differentiation; SRIR, self-recognizing inhibitory receptors; EBV, Epstein–Barr virus; CMV, cytomegalovirus; ALL, acute lymphoblastic leukemia; AML, acute myeloid leukemia; IFN, interferon; ILC, innate lymphoid cell; DCs, dendritic cells; RANTES, regulated upon activation, normal T-cell expressed, and secreted; CCL5, chemokine ligand 5; GM-CSF, granulocyte-macrophage colony-stimulating factor; TNF-α, tumor necrosis factor alpha; HSCT, hematopoietic stem cell transplantation; ROS, reactive oxygen species; CMCC, chronic mucocutaneous candidiasis; SLE, systemic lupus erythematosus; HIV, human immunodeficiency virus; IL, interleukin; TRAIL, tumor necrosis factor related apoptosis inducing ligand; IgG, immunoglobulin G; NADPH, nicotinamide adenine dinucleotide phosphate; Hsp, heat shock protein; NCR, natural cytotoxicity receptors; Gr-MDSC, granulocyte myeloid-derived suppressor cell; ITAM, immunoreceptor tyrosine-based activation motif.
